# Compliance of clinical trial registries with the World Health Organization minimum data set: a survey

**DOI:** 10.1186/1745-6215-10-56

**Published:** 2009-07-22

**Authors:** Lorenzo P Moja, Ivan Moschetti, Munira Nurbhai, Anna Compagnoni, Alessandro Liberati, Jeremy M Grimshaw, An-Wen Chan, Kay Dickersin, Karmela Krleza-Jeric, David Moher, Ida Sim, Jimmy Volmink

**Affiliations:** 1 Italian Cochrane Centre, Mario Negri Institute for Pharmacological Research, Milan, Italy; 2 Clinical Epidemiology Program, Ottawa Health Research Institute, Canada; 3 Università degli Studi di Modena e Reggio Emilia, Modena, Italy; 4 Institute of Population Health, University of Ottawa, Canada; 5 Department of Medicine, University of Toronto, Canada; 6 Randomised Controlled Trials Unit, Canadian Institutes of Health Research, Ottawa, Canada; 7 Johns Hopkins Bloomberg School of Public Health, Baltimore, USA; 8 Chalmers Research Group, Children's Hospital of Eastern Ontario Research Institute; Department of Epidemiology & Community Medicine, Faculty of Medicine, University of Ottawa, Canada; 9 Department of Medicine, University of California, San Francisco, USA, and World Health Organization, Geneva, Switzerland; 10 Faculty of Health Sciences, University of Stellenbosch, Cape Town and South African Cochrane Centre, Medical Research Council, South Africa

## Abstract

**Background:**

Since September 2005 the International Committee of Medical Journal Editors has required that trials be registered in accordance with the World Health Organization (WHO) minimum dataset, in order to be considered for publication. The objective is to evaluate registries' and individual trial records' compliance with the 2006 version of the WHO minimum data set.

**Methods:**

A retrospective evaluation of 21 online clinical trial registries (international, national, specialty, pharmaceutical industry and local) from April 2005 to February 2007 and a cross-sectional evaluation of a stratified random sample of 610 trial records from the 21 registries.

**Results:**

Among 11 registries that provided guidelines for registration, the median compliance with the WHO criteria were 14 out of 20 items (range 6 to 20). In the period April 2005–February 2007, six registries increased their compliance by six data items, on average. None of the local registry websites published guidelines on the trial data items required for registration. Slightly more than half (330/610; 54.1%, 95% CI 50.1% – 58.1%) of trial records completed the contact details criteria while 29.7% (181/610, 95% CI 26.1% – 33.5%) completed the key clinical and methodological data fields.

**Conclusion:**

While the launch of the WHO minimum data set seemed to positively influence registries with better standardisation of approaches, individual registry entries are largely incomplete. Initiatives to ensure quality assurance of registries and trial data should be encouraged. Peer reviewers and editors should scrutinise clinical trial registration records to ensure consistency with WHO's core content requirements when considering trial-related publications.

## Background

Registering clinical trials is a topical issue for the health research community.[1] More than 30 years ago, the first trials registry was initiated as a way to keep track of all trials initiated and to make possible retrieval of information about unpublished trials.[2] In the interim, several trials registries have emerged, for many different purposes, including recruiting patients to trials.[3]

To those performing systematic reviews, trials registries provide an essential tool to assess completeness of the information about all initiated trials addressing a given research question, regardless of a trial's ultimate publication status. But to judge a trial's eligibility for inclusion in a systematic review, the review team needs access to key protocol information. A "minimum dataset" was initially proposed in 1993[4] and has been updated since then.[5] Despite a proliferation of small, specialized registries and interest in a more comprehensive approach, there is currently no single worldwide registry that contains all ongoing trials and is considered the acknowledged repository of trial data. Coordinated efforts to assure global trial registration have lagged until recently, when a series of events caught the attention of the broader medical community.

In 2004 the International Committee of Medical Journal Editors (ICMJE), responding to reporting failures related to harms from anti-depressants [6], strongly encouraged the registration of trials. They issued a policy requiring trials commencing participant enrollment after September 2005 to be registered in order to be considered for publication within their journals.[7] The ICMJE does not mandate registration in any particular registry as long as it is electronically searchable, freely accessible to the public, open to all registrants, and managed by a non-profit organization. However, the ICMJE requires that trials registration adhere to the 20-item minimum dataset defined by the World Health Organization (WHO).[5] The World Association of Medical Editors supported the ICMJE campaign to register all clinical trials at their inception.[8] The objective of this descriptive study is to evaluate whether trial registries and individual trial records within the selected trial registries complied with the WHO minimum data set drafted in April 2005[5], issued in February 2006[9].

## Methods

We defined a trial registry as a database of planned, ongoing or completed trials, published or unpublished, containing details of the trial's objectives, patient population, sample size, and tested interventions.[4] This definition is in agreement with the definition by the WHO.[10] An official entry in the registry for a single trial is referred to as a trial record.

### Registry Compliance

In April 2005, we selected a convenience sample of 21 trials registries for this study. These registries included *international, national, disease specific, pharmaceutical industry*, and *local registries*. Details of the registries are presented in Additional File 1. The registries were chosen because they were widely known (except for the local registries), online, active, freely accessible, and in English. To select local registries we randomly sampled 10 institutions out of 184 entities referring to limited geographic area (e.g. UCSF University of California at San Francisco) and listed in the 'hospitals and clinical research centres' subset of TrialsCentral.[11]

For each registry in our sample, two reviewers independently abstracted whether key protocol items were present. We defined key protocol items as the 20-item WHO minimum dataset drafted in April 2005 [5] and the subsequent version updated in February 2006 [9], and their definitions for the appraisal checklist (Table 1). We assessed how many of the WHO criteria were available for registrants to complete (*'registry compliance with WHO criteria'*). Two members of the team independently (LPM, MN) continued to monitor trial registries' websites from April 2005 to February 2007, collecting information included on data fields, amendments and additions and any mention of the ICMJE or WHO initiatives about registration. Disagreements were resolved by discussion between the two study authors. We used a cohort design to evaluate trial registry compliance over a prolonged timeframe (number of WHO items included in each registry between April 2005 and February 2007).

**Table 1:** WHO minimal dataset: version issued in April 2005 (used as checklist in this study) [5] and revised version issued in February-March 2006[9]

	**Item 2005**	**Revised Item 2006**	Abridged Definition/Explanation*
1	Unique trial number	Primary Registry and Trial Identification number	Name of Primary Registry, and the unique ID number assigned by the Primary Registry to this trial.

2	Trial registration date	Date of Registration in Primary Registry	Date when trial was officially registered in the Primary Registry.

3	Secondary IDs	Secondary identification number(s)	Other identifying numbers and issuing authorities besides the Primary Registry, if any.

4	Funding source(s)	Source(s) of Monetary or Material Support	Major source(s) of monetary or material support for the trial (e.g., funding agency, foundation, company).

5	Primary sponsor	Primary Sponsor	The individual, organization, group or other legal person taking responsibility for securing the arrangements to initiate and/or manage a trial (including arrangements to ensure that the trial design meets appropriate standards and to ensure appropriate conduct and reporting).

6	Secondary sponsor(s)	Secondary Sponsor(s)	Additional individuals, organizations or other legal persons, if any, that have agreed with the primary sponsor to take on responsibilities of sponsorship.

7	Responsible contact person	Contact for Public Queries	Email address, telephone number, or postal address of the contact who will respond to general queries, including information about current recruitment status

8	Research contact person	Contact for Scientific Queries	Email address, telephone number, or postal address, and affiliation of the person to contact for scientific queries about the trial.

9	Title of the study (brief title)	Public Title	Title intended for the lay public in easily understood language.

10	Official scientific title of the study	Scientific Title	Scientific title of the trial as it appears in the protocol submitted for funding and ethical review. Include trial acronym if available.

11	Research ethics review		Eliminated
	
		Countries of Recruitment	The countries from which participants will be, are intended to be, or have been recruited.

12	Condition	Health Condition(s) or Problem(s) Studied	Primary health condition(s) or problem(s) studied (e.g., depression, breast cancer, medication error).

13	Intervention(s)	Intervention(s)	Enter the specific name of the intervention(s) and the comparator/control(s) being studied. Use the International Non-Proprietary Name if possible. If the intervention consists of several separate treatments, list them all. For each intervention, describe other intervention details as applicable (dose, duration, mode of administration, etc).

14	Key inclusion and exclusion criteria	Key Inclusion and exclusion criteria	Inclusion and exclusion criteria for participant selection, including age and sex.

15	Study type	Study Type	A single arm trial is one in which all participants are given the same intervention. A trial is "randomized" if participants are assigned to intervention groups using a method based on chance.

16	Anticipated trial start date	Date of First Enrollment	Anticipated or actual date of enrollment of the first participant.

17	Target sample size	Target Sample Size	Number of participants that this trial plans to enroll.

18	Recruitment status	Recruitment Status	Recruitment status of this trial (e.g., pending, active, temporary halt, closed).

19	Primary outcome	Primary Outcome(s)	Outcomes are events, variables, or experiences that are measured because it is believed that they may be influenced by the intervention. The Primary Outcome should be the outcome used in sample size calculations, or the main outcome(s) used to determine the effects of the intervention(s). Enter the names of all primary outcomes in the trial as well as the pre-specified timepoint(s) of primary interest.

20	Key secondary outcomes	Key Secondary Outcomes	Secondary outcomes are events, variables, or experiences that are of secondary interest or that are measured at timepoints of secondary interest.

* From the final version released in February 2006

### Record Compliance

From each of the 21 selected registries we randomly sampled a convenience preset number of trial records, to reach a planned total of 600 records (final sample of 610 single trial records). Samples varied according to registry storage. No restrictions were placed on trial status, design or medical area, although some registries had adopted inclusion criteria. The trial record data collection was completed between April and August 2005.

We assessed how commonly the WHO criteria were actually completed in single trial records (*'record compliance'*). When necessary, specific items were operationalized: for example, the WHO criteria 'contact details' was divided into 'name of contact person' and 'address', 'telephone', 'fax' or 'e-mail'. Key clinical and methodological details were defined as the presence of condition, intervention, study type, at least one outcome and key inclusion and exclusion criteria. Although different registries and guidelines define the minimum data items with varying levels of quality and detail, our operational definitions for considering an item "*compliant*" were inclusive. For example, an intervention item that reports "subjects will be assigned into either cyclophosphamide (0.5 to 1 g/m^2^) or methylprednisolone (1 g/m^2^) infusion; both treatments will be administered every four weeks during one year" would have been considered completed in the trial record for the purpose of this study, whereas an intervention reported as "adjuvant treatment" without other details would have been considered incomplete (See Additional file 2 for the operational definitions we adopted). Duplicate data extraction was undertaken for the first 50 trial records and inter-rater reliability was assessed with k statistic (0.80–1 k statistic values for the majority of fields). Subsequent data extraction was undertaken by a single rater. We calculated 95% confidence intervals using binomial approximation.

The evaluation of the 610 individual trial records was done at a single point in time (April-August 2005) and thus is a cross-sectional study.

Results are presented as percentage compliance with WHO items. The percentages are expressed with 95% confidence intervals in parentheses. Percentage of registry not compliant items for 2005 and 2006 were calculated as independent data and the difference between the two percentages were evaluated by Chi-Square test.

## Results

### Registry Compliance

Table 2 lists registries and compliance by item with the WHO minimum data set. None of the local registry websites published definitions of the trial information (submission fields) required for registration or mentioned the recent initiatives of the ICMJE or WHO; two local registries did not include any trial records.

**Table 2:** Types of trial registries and compliance with WHO criteria at the end of data collection period (February 2007).

	**Registries**												
	International		National		Specialty			Pharma				Local	Total out of 11 registries (excluding locals)
**Criteria requested**	ISCRTN	CT	ACTR	UK NRR	PDQ	STD	RT^†^	R	GSK	N	CSR		
Details not presented by registries												**x**	

Details available													
Unique trial number	x	x	x	x	x	x		x		**x**	**x**		**9**

Trial registration date	x	**x**	x					**x**					**4**

Secondary Ids	x	**x**	x	x	x						**x**		**6**

Funding source(s)	x	x	x	x	x	x	x		x	**x**	**x**		**10**

Primary sponsor	x	**x**	x	x	x		x	x			**x**		**8**

Secondary sponsor(s)	x	**x**	x	x				X			**x**		**6**

Responsible contact person	x	x	x			**PNR**		x					**5**

Research contact person	x	x	x	x	x	x	x				**x**		**8**

Title of the study (brief title)	x	x	x		x	x		x		**x**	**x**		**8**

Official scientific title of the study	x	x	x	x	x	x	x	x	x		**x**		**10**

Research ethics review*	**x**	**PNR**	x	**MC**	**PNR**								**4**

Countries of recruitment (replaced ethics review, May 2006)*	**x**	x	**x**	x	x			**x**					**6**

Condition	x	x	x	x	x	x		x	x	**x**	**x**		**10**

Intervention(s)	x	x	x		x	x		x	x	**x**	**x**		**9**

Key inclusion and exclusion criteria	x	x	x		x	x		x	x		**x**		**8**

Study type	x	x	x	x		x		x	x	**x**			**8**

Anticipated trial start date	x	**x**	x	x	x	x		**x**	x		**x**		**9**

Target sample size	x	**x**	x		x	x			x		**x**		**7**

Recruitment status	**x**	x	x		x	x	x	x					**7**

Primary outcome	x	**x**	x	x	x	x		**x**	x		**x**		**9**

Key secondary outcomes	x	**x**	x	x		x		**x**	x				**7**

**Total out of 20 criteria^‡^**	**20**	**20**	**20**	**13**	**16**	**14**	**5**	**16**	**10**	**6**	**14**		

Mentions WHO 2005/2006	**x**	**x**	**x**	**x**	**-**	**x**	**-**	**-**	**-**	**-**	**-**		

Mentions ICJME 2004/2005	**x**	**x**	**x**	**x**	**-**	**-**	**-**	**-**	**-**	**-**	**-**		

X = items present when data collection begun; **X **= items added during study period; ^‡^Total criteria considering the field 'Countries of recruitment'.

MC = only for multi-centre trials; PNR = provided not reported.

Abbreviation: ISRCTN, Current Controlled Trials; CT, ClinicalTrials.gov; ACTR, Australian Clinical Trials Registry; UK NRR, UK National Research Register; PDQ, US National Cancer Institute; STD, Stroke Trials Directory; RT, Rehabilitation Trials (^† ^access to web site withdrawn in 2006); R, Roche; GSK, GlaxoSmithKline; N, Novartis; CSR, ClinicalStudyResults.

During the period April 2005 to February 2007, six registries increased their compliance with WHO criteria by 6 data items, on average, ameliorating their compliance from 10 to 16 items. Two international (ClinicalTrials.gov and ISRCTN) and one national registry (ACTR) modified their content submission fields to become fully compliant with WHO standards during our data collection period. Two pharmaceutical industry registries (Novartis and ClinicalStudyResults) that completely lacked registration criteria and definitions in April 2005 modified their structure, increasing the number of items offered to 6 and 14 respectively. The Roche registry rose from 9 to 16 items. The RehabTrials.org registry stopped being accessible in 2006. In the subgroup of registries providing guidelines for registration (11 registries), by February 2007, median compliance with the WHO criteria was 14 out of 20 items (range 6 to 20). From 2005 to 2006 the number of registry not compliant items decreased significantly from 46.4% (39.6–53.2) to 30.0% (24.0–36.5) (p = 0.0039).

### Record Compliance

**Figure 1:**
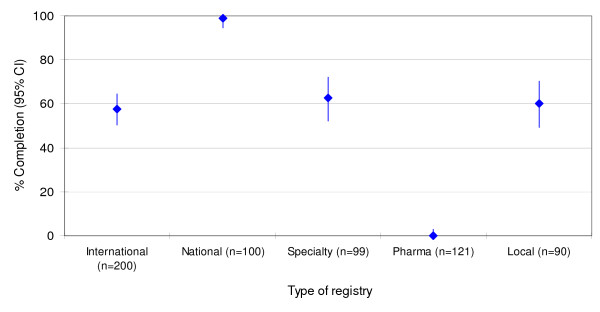
**Contact details**. Percentage (95% confidence interval) of trial records reporting minimum contact details (defined as the presence of name of contact person and one additional item: address, telephone, fax or e-mail) by type of registry.

**Figure 2:**
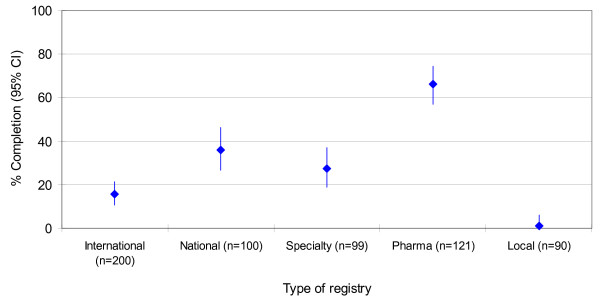
**Clinical and methodological details**. Percentage (95% confidence interval) of trial records reporting clinical and methodological details (defined as the presence of condition, intervention, study type, at least one outcome and key inclusion and exclusion criteria) by type of registry.

Table 3 lists the percentages of compliant records relative to registries. The final sample of 610 trial records covered different years (range: 1981 to 2005). However the majority of trials were recently registered (<1999 n = 77 (13% out of 610); 2004–2000 n = 246 (40% out of 610); 2005 n = 156 (26% out of 610)). In 131 records the registration date was not available. Overall, 330 trial records, 54.1% (50.1% – 58.1), completed the contact details criteria. Trial records in national registries adhered more often with this requirement (compliance 99%) while those in drug company registries never reported it (Figure 1). 181 records, 29.7% (26.1 – 33.5), provided complete information about key aspects of trial design (target condition, intervention, study type, at least one outcome and key inclusion and exclusion criteria; Figure 2). Among these five key methodological data fields compliance varied across items from 40.5% (36.6 – 44.5) for primary outcome measures to 75.2% (71.6 – 78.6) for target condition. 'Research ethics review' (6.9% (5.0% – 9.2)), 'responsible contact person' (8.2% (6.1 – 10.7)) and 'secondary outcomes' (21.6% (18.4% – 25.1)) had lower compliance rates.

**Table 3:** Percentage of compliance of trial records according to WHO criteria by trial registry.

	**Registries (number of records)**												
	International		National		Specialty			Pharma					

Criteria requested	ISCRTN (n = 100)	CT (n = 100)	ACTR (n = 50)	UK NRR (n = 50)	PDQ (n = 33)	STD (n = 33)	RT (n = 33)	R (n = 8)	GSK (n = 72)	N (n = 4)	CSR (n = 37)	Local (n = 90)	Total, % (95% CI)

Unique trial number	100	100	100	100	0	0	0	100	100	100	97.3	90	82.1 (78.9 to 85.1)

Trial registration date	100	100	100	100	100	100	100	100	100	0	0	0	78.5 (75.0 to 81.7)

Secondary Ids	100	91	16	4	100	0	0	0	0	0	0	0	38.4 (34.5 to 42.4)

Funding source(s)	100	100	96	76	66.7	69.7	90	100	100	100	100	27.8	83.1 (79.9 to 86.0)

Primary sponsor	100	100	90	92	66.7	69.7	90.9	100	31.9	100	100	27.8	75.9 (72.3 to 79.2)

Secondary sponsor(s)	14	10	38	0	0	6.1	6.1	0	0	0	0	0	7.7 (5.7 to 10.1)

Responsible contact person	0	0	100	0	0	0	0	0	0	0	0	0	8.2 (6.1 to 10.7)

Research contact person	95	20	100	98	93.9	63.6	30.3	0	0	0	0	60	54.1 (50.1 to 58.1)

Title of the study (brief title)	98	18	66	0	0	60.6	0	0	0	0	0	12.2	29.5 (25.9 to 33.3)

Official scientific title of the study	83	95	100	94	97	69.7	84.9	100	100	0	89.2	82.2	89.3 (86.6 to 91.7)

Research ethics review	1	0	78	0	3	0	3	0	0	0	0	0	6.9 (5.0 to 9.2)

Condition	70	85	92	66	97	100	84.9	100	95.8	100	83.8	22.2	75.2 (71.6 to 78.6)

Intervention(s)	56	68	74	24	75.8	97	42.4	0	100	0	64.9	17.8	58.4 (54.3 to 62.3)

Key inclusion and exclusion criteria	23	97	100	44	18.2	87.9	0	100	97.2	50	64.9	28.9	58.5 (54.5 to 62.5)

Study type	43	74	98	58	9.1	97	33.3	0	94.4	0	64.9	45.6	61.3 (57.3 to 65.2)

Anticipated trial start date	8	65	100	100	0	39.4	3	0	100	0	64.9	0	46.4 (42.4 to 50.4)

Target sample size	40	83	100	24	72.7	100	39.4	0	80.6	0	64.9	0	55.2 (51.2 to 59.2)

Recruitment status	3	99	100	100	93.9	100	12.1	100	0	0	54.1	7.8	50.0 (46.0 to 54.0)

Primary outcome	7	48	100	34	0	100	18.2	0	83.3	0	64.9	2.2	40.5 (36.6 to 44.5)

Key secondary outcomes	6	10	58	6	6.1	54.6	3	0	61.1	0	48.7	1.1	21.6 (18.4 to 25.1)

Note: In CT, PDQ and STD 'Funding source(s)' and 'Primary sponsor' are not distinct. By default contact person without specification has been refereed to Research contact person.

Abbreviation: ISRCTN, Current Controlled Trials; CT, ClinicalTrials.gov; ACTR, Australian Clinical Trials Registry; UK NRR, UK National Research Register; PDQ, US National Cancer Institute; STD, Stroke Trials Directory; RT, Rehabilitation Trials; R, Roche; GSK, GlaxoSmithKline; N, Novartis; CSR, ClinicalStudyResults.

## Discussion

### Summary of key findings

As of February 2007, the compliance of information in trial registries is unsatisfactory despite the emerging consensus that the availability of such information is ethically and scientifically essential.[12] We found that in August 2005, only 54% of trial records provided adequate contact information and less than 30% contained the complete information necessary to provide a general picture of trial objectives, such as outcome measures and details of the intervention. The launch of the WHO minimum dataset and its enforcement by the ICMJE seemed to positively influence registries: 6 out of 11 increased their compliance by the time of the ICMJE requirement. Some of the WHO criteria seemed to be easily adopted by registries, while others were less so: compliance was variable among registries and there were inconsistencies between registry-offered fields and record compliance (i.e., many registries failed to offer all 20 WHO data items for completion, and registrants failed to comply with many of the data items that were offered).

### Strengths and weaknesses

Our assessment was limited to a sample of 21 registries to reflect different types of registries.[13] Our sample of records can be criticised in some respects. Although we chose the registries to reflect diversity based upon a *priori *defined important information (e.g., target health professionals or patients, profit or no-profit aims, etc), and without prior knowledge of what we would find, the selection was largely of registries published in English. We considered including registries from Spain and Italy which have country-wide mandatory trial registration [14,15] but their registries did not meet our inclusion criteria of being in the public domain. In Italy only funding agencies and ethics committees have unrestricted access. In Spain the online version of their national registry is still under construction. It is possible that these registries differ from others in terms of the amount and quality of information collected and this may limit the generalisability of this study. Our results for a sample of trials registries in 2005–2007 are a snapshot from what has become a rapidly evolving field. For example, in 2005–2007 the WHO formally established the International Clinical Trials Registry Platform (ICTRP) to standardise the scope and content of trial registration.[16] The WHO finalized the criteria to build a global network of qualified registries, adopting a hierarchical structure (primary and partner registries) and creating a web site that enables users to search a central database that contains the trial registration data sets provided by primary registries.[17] Triggered by the ICMJE and WHO initiatives, trial registration has become very active. Thus, ClinicalTrials.gov increased from a routine weekly registration of 30 new trials to 220 new trials [18] and changed its registration requirements .[19] ISRCTN's trial records expanded from 2 705 to 6 449, and transferred its ownership to a not-for-profit organization to comply with the ICMJE requirement that registries be non-profit adopting a new URL .[20] Since 2007, ISRCTN has required an administrative charge (£132) to registry new trials, while ClinicalTrials.gov maintains a free of charge policy. The Australian Clinical Trials Registry (ACTR) was established in April 2005 by merging old registries into a new highly standardised version incorporating the complete WHO minimum dataset.[21] At the time of our study, the Lilly website only linked to its records in ClinicalTrials.gov but has since launched its own registry with information about recruiting and non-recruiting trials, while also providing identification numbers.[22]

Another element of change is related to our assessment tool: we used the draft April 2005 WHO minimum dataset to assess the trials registered in our study, although the finalised version released in February 2006 was somewhat different (*research ethics review *item was removed and *Countries of recruitment *was added).[9] The draft and final dataset versions are shown in Table 1 (definitions/explanations are from the final version). Neither version of the WHO dataset was available for use as a benchmark by registries and registrants before April 2005. Our findings highlight the variation in registry and record compliance with the 2005 WHO criteria up to February 2007. It should be also stressed that registry and record compliance are not independent since registrants can only provide the information requested, and therefore the variation in registry compliance will constrain record compliance. As with previous studies, we could not evaluate the actual proportion of trials registered among all trials conducted over the time period examined: this could be achieved only assessing the number of trials launched at the source point (e.g., funding agencies, industry, ethics committees, regulators) and it is out of the scope of the present work.

### Our study in context

Our results partially overlap with the results reported by Zarin et al. who surveyed record completion on ClinicalTrials.gov between May and October 2005.[23] Zarin assessed completeness of *Intervention name *(compliance rates at 100% and 90% for non-industry and industry records, respectively) and *Primary outcome *(compliance rate available only for industry records, 76%). We found lower compliance for *Intervention name *(68%), though this difference could be because we examined registries over a different time interval. As Zarin et al. showed, many records changed their completion of registration around the time of ICJME deadline: it is possible that investigators were motivated to update the recent records over the older ones or that registries' editors started to scrutinise trial records with more stringent policies.[18] If true, this could also explain the lower compliance rate we found for *Primary outcome *compared to Zarin et al. Completion rate also depends on the operational definition adopted by assessors. The definition used by Zarin et al. is less stringent compared to the one we adopted.

An interesting result of our study is that industry registries appear to satisfy WHO minimal dataset in terms of methodological details more completely than non-industry registries. This result seems to contrast with the pharmaceutical industry's concern over the disclosure of the five methodological items.[24] This finding could be due to trials listed on company registries are for 'approved drugs' and the information is no longer considered to be commercially sensitive. Another possibility is that the different drug companies, while having a common overall position, ultimately adopt heterogeneous policies on disclosing their data items. Pharmaceutical company registries did not include details in their registries for a contact persons for each trial, although they did include an e-mail address for additional information about trials. This was not considered as meeting the WHO criterion, however, as accountability appeared too vague if a contact name was not provided. These results have been confirmed by another study which analysed the proportion of trial records listing complete contact information of Canadian investigators in a sample of records in ISRCTN and ClinicalTrials.gov and found largely incomplete contact information in industry funded trials.[25]

## Conclusion

### Implications for systematic reviewers

As part of their broad search to identify potentially eligible data systematic reviewers should include trial registries for ongoing trials, particularly in situations where there is great uncertainty about the efficacy of an intervention and it is possible that new trial data may influence the summary judgment of the review. Our findings revealed that registries often do not contain meaningful information on many key methodological data fields and thus at this time cannot be used reliably as referent information sources to describe included studies in systematic reviews. Details of research contact persons, when present, can be used to address questions about methodological aspects of a trial or unpublished data.

### Implications for trial registration

In the move towards global trial registration, there is room for better standardisation of approaches and better reporting of registration data items. This effort is in keeping with other global efforts to improve the reporting of randomised trials, such as the CONSORT Statement.[26] The WHO is developing criteria for internationally acceptable trial registries, and has established a working group of trial registries to develop better approaches to data entry validation and other aspects of quality assurance.[27] Given the variability in registry compliance and record completeness, editors and peer reviewers of medical journals should scrutinise trial registration records to ensure consistency with WHO's minimum dataset when considering trial-related publications and should report the trial identification numbers, including those assigned by WHO.

### Implications for research

As registries adopt the WHO minimum dataset, there is a need to assess the evolution of registries and records over time, and whether the 20 WHO criteria are sufficient to judge the scientific conduct of trials or should be expanded. Further research is also needed to determine whether early trial registration increases informed patient recruitment and improves quality and completeness of subsequent publications: the impetus for clinical trial registration stems from the added value of including all clinical trials, not just published ones, within systematic reviews.[6] As such, there is a need to prospectively monitor protocol amendments and the accessibility of unpublished clinical trial data. Including primary outcome information within a registry will also enable us to evaluate whether the disturbingly high frequency of outcome reporting bias declines.[28,29] Following from these ideas, trial registries will be most useful if they increase the accessibility of evidence, including data on adverse events. In other words, the real test for trial registries is whether they facilitate making the results of unpublished trials, and unpublished results of published trials, available to the public. whether through trial registries or dedicated results repositories or databases.[30] The United States FDA Amendments Act 2007 (U.S. Public Law 110-85), which is the world's first legislative requirement for the public reporting of trial results, is an important step in this direction. Simply registering trials is not going to solve the problem, but it is a necessary first step to enable identification of all trials and the subsequent tracking of their results.

Clinical trial registration was advocated more than thirty years ago[2], and important progress has recently been made. We have a scientific, ethical and moral obligation to clinical trial participants to ensure that clinical trial registries are created with and adhere to the highest possible standards.

## Competing interests

The authors declare that they have no competing interests.

## Authors' contributions

LM, AL and JMG conceived the study concept. LM, IM, MN, AL, JMG and AWC conceived the study protocol. LM, IM and MN were involved in the acquisition of data. LM, IM, MN and AC were involved in the analysis of data. LM, IM, MN, AL and JMG were involved in drafting of the manuscript. All authors were involved in the interpretation and critical revision of the manuscript for important intellectual content.

## Supplementary Material

Additional file 1 **Appendix 1**. URLs and type of trials registries. Accessed November 2005 and monitored until February 2007. Click here for file

Additional file 2 **Appendix 2**. Operational definitions adopted for the purposes of this study. Click here for file
