# Supplementary material for: Compliance of clinical trial registries with the World Health Organization minimum data set: a survey
Source: Trials. 2009 Jul 22;10:56. doi: 10.1186/1745-6215-10-56 (PMC2734552; doi:10.1186/1745-6215-10-56)
Supplement: Additional file 1 — Appendix 1. URLs and type of trials registries. Accessed November 2005 and monitored until February 2007. [file 1745-6215-10-56-S1.doc]

Appendix 1. URLs and type of trials registries. Accessed November 2005 and monitored until February 2007.

| **Register** | **Type** | **URL** |
| --- | --- | --- |
| ISRCTN (Current Controlled Trials) | International | http://www.controlled-trials.com/isrctn/ |
| ClinicalTrials.gov (U.S. National Institutes of Health) | International | http://clinicaltrials.gov/ct/gui |
| DH - Department of Health | National | http://www.update-software.com/national/ |
| ACTR Australian Clinical Trials Registry | National | http://www.actr.org.au/ |
| Stroke Trials Directory | Specialty | http://www.strokecenter.org/trials/browse.aspx |
| RehabTrials.org (access to web site withdrawn in 2006) | Specialty | http://www.rehabtrials.org/Trials/trial_list.shtml |
| National Cancer Institute | Specialty | http://www.cancer.gov/clinicaltrials |
| GlaxoSmithKline | Pharma | http://ctr.glaxowellcome.co.uk/MedicineList.asp |
| Roche | Pharma | http://www.roche-trials.com/ |
| Novartis | Pharma | http://www.novartisclinicaltrials.com/etrials/home.do |
| PhRMA | Pharma | <http://www.clinicalstudyresults.org/> |
| TrialsCentral | Database of registers | http://www.trialscentral.org/ClinicalTrials.aspx |
| AIDS Clinical Trials Unit (ACTU) at Indiana University | Local | http://www.aactg.org/clinicaltrials_actulocator.asp |
| Community Consortium: University of California at San Francisco | Local | http://medschool.ucsf.edu/clinical_trials/ |
| Comprehensive Cancer Center at the University of Michigan | Local | https://www.umms.med.umich.edu/engage/disp_pub_condition.do |
| Harvard Bipolar Research Program | Local | http://www.manicdepressive.org/currentstudies.php# |
| Integris Health | Local | <http://www.integris-health.com/INTEGRIS/en-US/default.htm> |
| North Shore-Long Island Jewish Health System | Local | http://www.northshorelij.com/bodyiframe.cfm?ID=1859 |
| University of California at San Francisco (UCSF) Cancer Center | Local | http://medschool.ucsf.edu/clinical_trials/ |
| University of Alabama Comprehensive Cancer Center | Local | http://www2.ccc.uab.edu/CSUWEB/ClinicalTrialsListing.asp |
| Winship Cancer Institute of Emory University | Local | http://cancer.emory.edu/clinical/ |
| Xavier University Clinical Trials Unit | Local | http://www.xula.edu/pharmacy/CTU/clinical-trials.htm |
