# Supplementary material for: Compliance of clinical trial registries with the World Health Organization minimum data set: a survey
Source: Trials. 2009 Jul 22;10:56. doi: 10.1186/1745-6215-10-56 (PMC2734552; doi:10.1186/1745-6215-10-56)
Supplement: Additional file 2 — Appendix 2. Operational definitions adopted for the purposes of this study. [file 1745-6215-10-56-S2.doc]

Appendix 2. Operational definitions adopted for the purposes of this study.

| Criteria requested | **Operational definitions** | **Example (where relevant)** |
| --- | --- | --- |
| Unique trial number | Identifier, ID number or code given by registry | ISRCTN68958618 |
| Trial registration date | Date of first registration of the trial (if present, record day/month/year) |  |
| Secondary IDs | Additional ID numbers or codes given by the trial sponsors and or other interested parties | A2631 |
| Funding source(s) | In CT, PDQ and STD ‘Funding source(s)’ and ‘Primary sponsor’ are not distinct. Record only if are clearly distinct from sponsors | External funding has been secured from the following organisations: Eli Lilly, Roche Products, Sanofi-Aventis |
| Primary sponsor | If more sponsors are listed consider checking both primary and secondary sponsors | ANZ Breast Cancer Trials Group Ltd. |
| Secondary sponsor(s) | If more sponsors are listed consider checking both primary and secondary sponsors |  |
| Responsible contact person | Responsible contact person is only for public queries |  |
| Research contact person | Research contact person is any contact, without specification about type of queries |  |
| Title of the study (brief title) | Acronym or short title of study | Capecitabine for Advanced Breast Cancer |
| Official scientific title of the study | Official scientific title of study should include name of intervention and condition being studied (eventually also the study type and outcome) | A randomised phase II study comparing the safety and efficacy of capecitabine with capecitabine and oral cyclophosphamide in patients with advanced breast cancer |
| Research ethics review | Number and or list of research ethics committees. This item was replaced by Countries of recruitment in May 2006 | Ethics committee of the Istituto Nazionale per lo Studio e la Cura dei Tumori (IRCCS), Milano, Italy |
| Countries of recruitment | List of countries of recruitment. States or provinces are acceptable. This item replaced Research ethics review in May 2006 |  |
| Condition | The medical condition being studied. Generic disease description such as ‘cancer’ without any specification is not acceptable | Metastatic breast cancer |
| Intervention(s) | Brief description of all interventions (or trial arms) and the duration of intervention including drug name or serial number for an unregistered drug. Generic intervention as pain therapy or pain control without detail on how it is delivered (e.g. dose and timing) is not acceptable | Low dose aspirin (100mg daily)  Statin 80mg for 21 days  12-week physical exercise program |
| Key inclusion and exclusion criteria | Eligibility criteria (patient characteristics and key inclusion - exclusion criteria); The presence of only one, inclusion or exclusion criteria, is accepted | Inclusion: Histological or cytological evidence of breast carcinoma with at least one of the following: distant metasteses, T4 or N2 or N3, or loacal recurrence following mastectomy;  Exclusion: pregnancy or women of child-bearing potential who are at risk of pregnancy |
| Study type | Design features as RCT, parallel, cross-over  Experimental features as interventional or observational  Note: Study type information can often be found in other record fields | A randomised, double-blind, placebo-controlled trial |
| Anticipated trial start date | Enrolment date of the first patient (or its estimate) (if present, record day/month/year) |  |
| Target sample size | Total number of participants planned for enrolment |  |
| Recruitment status | Current trial status  e.g. ongoing, number of patients recruited or closed to recruitment | Open to recruitment |
| Primary outcome | Primary outcome that study was designed to evaluate (description should include time of measurement or duration of follow up). Generic outcome such as “response” for cancer or “depression” without specification of how it is measured (name of scale or type of index) is not acceptable | Overall survival – five years follow up  Overall response – one year follow up  Pain as assessed by visual analogue scale at 1 hour |
| Key secondary outcomes | Secondary outcome(s) specified in the protocol (description should include time of measurement or duration of follow up). Generic outcome such as “response” for cancer or “depression” without specification of how it is measured (name of scale or type of index) is not acceptable | Overall survival – five years follow up  Overall response – one year follow up  Pain as assessed by visual analogue scale at 1 hour |
